# Vanadium Oxide-Poly(3,4-ethylenedioxythiophene) Nanocomposite as High-Performance Cathode for Aqueous Zn-Ion Batteries: The Structural and Electrochemical Characterization

**DOI:** 10.3390/nano12213896

**Published:** 2022-11-04

**Authors:** Filipp S. Volkov, Svetlana N. Eliseeva, Mikhail A. Kamenskii, Alexey I. Volkov, Elena G. Tolstopjatova, Oleg V. Glumov, Lijun Fu, Veniamin V. Kondratiev

**Affiliations:** 1 Institute of Chemistry, Saint Petersburg State University, 7/9 Universitetskaya Nab, 199034 Saint Petersburg, Russia; 2 State Key Laboratory of Materials-Oriented Chemical Engineering, Nanjing Tech University, Nanjing 211816, China

**Keywords:** aqueous zinc-ion battery, vanadium oxide, cathode, poly(3,4-ethylenedioxythiophene), composite, energy storage, electrochemical performance

## Abstract

In this work the nanocomposite of vanadium oxide with conducting polymer poly(3,4-ethylenedioxythiophene) (VO@PEDOT) was obtained by microwave-assisted hydrothermal synthesis. The detailed study of its structural and electrochemical properties as cathode of aqueous zinc-ion battery was performed by scanning electron microscopy, energy dispersive X-ray analysis, X-ray diffraction analysis, X-ray photoelectron spectroscopy, thermogravimetric analysis, cyclic voltammetry, galvanostatic charge–discharge, and electrochemical impedance spectroscopy. The initial VO@PEDOT composite has layered nanosheets structure with thickness of about 30–80 nm, which are assembled into wavy agglomerated thicker layers of up to 0.3–0.6 μm. The phase composition of the samples was determined by XRD analysis which confirmed lamellar structure of vanadium oxide V_10_O_24_∙12H_2_O with interlayer distance of about 13.6 Å. The VO@PEDOT composite demonstrates excellent electrochemical performance, reaching specific capacities of up to 390 mA∙h∙g^−1^ at 0.3 A∙g^−1^. Moreover, the electrodes retain specific capacity of 100 mA∙h∙g^−1^ at a high current density of 20 A∙g^−1^. The phase transformations of VO@PEDOT electrodes during the cycling were studied at different degrees of charge/discharge by using ex situ XRD measurements. The results of ex situ XRD allow us to conclude that the reversible zinc ion intercalation occurs in stable zinc pyrovanadate structures formed during discharge.

## 1. Introduction

The growing demand for electrochemical power sources in modern technologies defines the development of stable and safe electrochemical devices. Nowadays, lithium-ion batteries are still the most widely used electrochemical source of energy, owing to their high energy density [1,2]. However, their drawbacks, including safety issues and decreasing availability and high price of lithium, dictate the need for the development of alternative metal-ion rechargeable batteries. Among multiple different types of batteries, aqueous zinc-ion batteries (AZIBs) have attracted increasing attention due to their high safety, environmental friendliness, low cost, and high gravimetric and volumetric capacities of metal zinc anode (820 mA∙h∙g^−1^ and 5855 mA∙h∙cm^−3^) [3,4].

Overcoming low cycling stability and rate capability of cathode materials for AZIBs is a major challenge in their successful implementation [3,5,6]. In the past decade since the first reports on AZIBs with mild electrolytes [7,8] employing MnO_2_ cathodes, many other types of cathode materials were proposed [9,10] including vanadium oxides [11,12], Prussian blue analogues [13], transitional metal disulfides [14], and organic compounds [15,16].

Of these, vanadium pentoxide V_2_O_5_ has the theoretical capacity value of up to 589 mA∙h∙g^−1^ per two-electron process [17], while most reported experimental results are ca. 300 mA∙h∙g^−1^ [9,18]. Further, layered-type structure of these materials allows high reversibility of ion intercalation [19]. Nevertheless, one of the major problems affecting the performance of vanadium oxide-based cathode materials is their dissolution [11,18], which leads to unsatisfactory performance, especially at lower current densities. In addition, highly crystalline vanadium oxide-based materials require activation of the material [20,21], which hinders the performance in the initial cycles.

Several strategies exist to overcome these drawbacks. These include nanostructuring [22], addition of conducting polymers [20,23] or carbons [24,25] as components or coatings, and inclusion of different metal ions into the crystal structure of the material via pre-intercalation [25,26] or manufacturing of pillared structures, i.e., the solids with the interlayer distance permanently expanded by inserting a strongly bound guest ion or molecule [9,27,28,29].

First, synthesis conditions affect the crystal lattice and regulate the amount of crystallized water in the molecule. Water molecules inserted into the crystal lattice can “lubricate” intercalation by regulating the interlayer distance [30,31] and can then be replaced by zinc ions during cycling [32]. It has been established that the presence of water in, e.g., V_2_O_5_·nH_2_O [33], NaCa_0.6_V_6_O_16_·3H_2_O [34], ZnMn_2_O_4_·0.94H_2_O [35], decreases the interaction energy of zinc ions with the crystal lattice, thus promoting the intercalation processes. Further, during recharging, co-intercalation of Zn^2+^ and water molecules facilitates the zinc ions diffusion [36].

The use of conductive additives, markedly conducting polymers along with traditional carbon additives, can significantly increase the electronic and ionic conductivity of composite materials and improve the diffusion of zinc ions inside the crystal lattice. These benefits arise from the formation of highly porous structures and reducing the coulombic interactions between zinc ions and lattice oxygen anions, via optimized local charge distribution in the crystal lattice [37]. Furthermore, chains or blocks of conducting polymer can be inserted into the crystal lattice and act as “pillars” enlarging the interlayer spacing and greatly enhancing its structural stability. This effect of interfacial engineering was described for V_2_O_5_ composites with polyaniline [38] and poly(3,4-ethylenedioxythiophene) PEDOT [39]. The application of an aniline-based polymer 3-phenylpropylamine led to enlarging of the interlayer distance to 18 Å [40]. The use of PEDOT also enhances the electrochemical properties of vanadium oxide- [41,42] and ammonium vanadate-based [43] electrode materials. The incorporation of PEDOT via in situ chemical approach with partial reduction of V_2_O_5_ in the presence of EDOT monomer may enhance the electrochemical performance by transformation of the initial structure into mixed-valence V_10_O_24_∙12H_2_O in the resulting vanadium oxide/conducting polymer composite [44].

A widely discussed problem is establishing the mechanism of electrochemical reaction occurring with vanadium oxide-based electrodes in mild zinc-based electrolytes [6,16,18,31,33]. Three possible pathways of reaction are discussed in the literature: traditional Zn^2+^ (de)intercalation [6,9], dual co-insertion of Zn^2+^ and H^+^ (or H_3_O^+^) [6,45], and phase transformation during the cycling process [31]. Ex situ XRD analysis confirmed the dual H^+^/Zn^2+^ insertion and formation of Zn_4_(OH)_6_SO_4_·4H_2_O for calcium vanadium oxide bronze cathodes [46], and in situ measurements for V_3_O_7_·H_2_O [47] and NaV_3_O_8_·1.5H_2_O [45]. For the anhydrous V_2_O_5_, water-accelerated vanadium dissolution has been reported, and cathodic formation of hydrated oxide was observed by ex situ and in situ XRD and SEM characterization [31]. Besides creation of “pillars” [23], the attempts to suppress dissolution include the addition of Na_2_SO_4_ salt to the electrolyte [45] or creating artificial solid electrolyte interphases (SEI) [48].

In this work, we performed a detailed study of structural and electrochemical properties of a cathode material based on vanadium oxide with conducting polymer. The vanadium oxide/PEDOT nanocomposite (VO@PEDOT) was obtained by microwave-assisted synthesis adopted from [49]. This method of synthesis was earlier used for the preparation of cathode materials for lithium-ion batteries [49], but the study of this kind of composite as a cathode in AZIBs was not performed previously. A facile one-step method at temperature 120 °C produced the composite cathode material with enhanced electrochemical performance. Its advantage over conventional hydrothermal methods is the minimal time required for the complete synthesis. The obtained nanocomposite demonstrated outstanding specific capacity, rate capability, and cyclic stability in the (0.3–1.4) V (vs. Zn/Zn^2+^) potential range. The high electrochemical performance of the VO@PEDOT electrodes was attributed to the formation of nanostructured powder with layered nanosheets structure with thickness of about 30–80 nm, which are assembled into wavy agglomerated more thicker layers up to 0.3–0.6 μm. The phase composition of the samples was determined by XRD analysis, which confirmed lamellar monoclinic V_10_O_24_∙12H_2_O structure with interlayer distance of about 13.6 Å. The mechanism of intercalation processes in the obtained material was systematically studied by ex situ XRD analysis. This allows us to show that initial structure can transform into layered pyrovanadate structure upon Zn^2+^ intercalation. The functional properties of the obtained cathode material were evaluated by cyclic voltammetry (CV), galvanostatic charge/discharge (GCD) to show applicability in AZIBs. Additionally, the analysis of the results obtained by EIS also presents novelty for the study of vanadium oxide-based composites and provides information on the beneficial effect of a conducting polymer on the charge transfer resistance in composite cathode and on the mass transport kinetics. We have confirmed that the apparent diffusion coefficient for VO@PEDOT is ca. 30 times higher than that of V_2_O_5_, which manifests itself in faster ion intercalation process for VO@PEDOT electrode. The effect of a conducting polymer on the vanadium oxide dissolution suppression was also elucidated.

## 2. Materials and Methods

### 2.1. Synthesis of VO@PEDOT Composite

The nanocomposite of vanadium oxide (V_10_O_24_) with conducting polymer poly(3,4-ethylenedioxythiophene) was synthesized by soft reduction of commercial V_2_O_5_ (NevaReaktiv LLC, Saint-Petersburg, Russia) by EDOT (97%, Sigma-Aldrich Corp., Burlington, MA, USA) under microwave radiation according to the procedure adopted from [49]. About 1.1 mmol of V_2_O_5_ was mixed with 0.42 mmol of EDOT in aqueous solution in a Pyrex reactor (35 mL). The resulting mixture was kept at 120 °C and 0.421 MPa for 1 h at 290 W microwave power level in a lab microwave system Discover SP (CEM Corp., Matthews, NC, USA). Following that, the obtained dark blue powders were washed with water and ethanol, and dried at 80 °C for 2 h. For the sake of simplicity, further the composite will be denoted as VO@PEDOT.

### 2.2. Characterization Methods

The powders of the pristine V_2_O_5_ and VO@PEDOT composite were characterized by X-ray diffraction (XRD) on a D8 DISCOVER spectrometer (Bruker AXS GmbH, Karlsruhe, Germany) using Cu-K_α_ radiation. Scanning electron microscopy (SEM) and energy dispersive X-ray analysis (EDX) were performed on powders on a SUPRA 40VP electron microscope (Carl Zeiss AG, Oberkochen, Germany) and Inca X-Act EDX spectrometer (Oxford Instruments GmbH, Wiesbaden, Germany), respectively.

Ex situ high-resolution (HR)-XRD studies of the electrode materials were performed after the electrochemical cycling. After extraction of the electrodes from the cells, they were carefully rinsed with DI water and dried in air. The thermogravimetric analysis (TGA) of a VO@PEDOT powder was performed in the (30–600) °C temperature range at a 10 °C/min heat rate in air on a TG 209 F1 Libra thermogravimetric analyzer (NETZSCH-Gerätebau GmbH, Selb, Germany). The X-ray photoelectron spectra (XPS) were measured on the Escalab 250Xi spectrometer (Thermo Fisher Scientific Inc., Waltham, MA, USA) equipped with an Al Kα source (photon energy of 1486.6 eV). X-ray photoelectron spectroscopy was used to study the states of the elements in the VO@PEDOT before and after electrochemical charge–discharge cycling. The spectra were recorded in the constant pass energy mode at 50 eV for the element core-level spectra and 100 eV for the survey spectra using the X-ray spot size of 650 μm. The overall experimental energy resolution was 0.3 eV. To counter the surface charge caused by the emitting photoelectrons, dual-mode charge compensation (a combination of low-energy electrons and argon ions) was used. XPS measurements were carried out at room temperature under UHV (around 1·10^−9^ mbar). The spectra were fitted with a product of asymmetric Gaussian and Lorentzian curves.

Comparative study of the solubility of the electrodes based on VO@PEDOT and pristine V_2_O_5_ was performed by inductively coupled plasma atomic emission spectroscopy (AES-ICP) on a ICP Emission Spectrometer ICPE-9000, (Shimadzu Corp., Kyoto, Japan). 1.5 mg electrode samples (in respect to vanadium oxide active material) of V_2_O_5_ and VO@PEDOT were submerged into 15 mL of zinc sulfate solution. The 1 mL samples of the solution were extracted in the first, second, third, fifth, and tenth days after immersion. The samples were diluted 18 times (mainly to decrease Zn sulfate contents) with deionized water, and then the elemental content of vanadium was studied using atomic emission spectroscopy. The calibration was performed for the solutions containing 0.01 mg∙dm^−3^ to 10 mg∙dm^−3^ of vanadium species. To estimate the long-term stability of the samples, another sample was extracted and analyzed six months later.

### 2.3. Electrochemical Characterization

Electrode materials were prepared by mixing the pristine V_2_O_5_ and VO@PEDOT with carbon black and polyvinylidene fluoride (PVDF) in a 70:20:10 weight ratio in N-methylpyrrolidone. The resulting viscous slurry was applied on the titanium foil (current collector), vacuum-dried at 60 °C, and roll-pressed. The mass loading of the electroactive material was 1.5–2.0 mg cm^−2^. Coin cells CR2032 were assembled vs. Zn anode with aqueous 3 mol dm^−3^ ZnSO_4_ (JSC LenReactiv, Saint-Petersburg, Russia) as electrolyte and Whatman^®^ glass fiber GF/A as separator.

The potentials in this work are referred to as Zn/Zn^2+^ redox pair unless stated otherwise. Electrochemical performance of the electrodes was studied using galvanostatic charge/discharge (GCD) and cyclic voltammetry (CV) in the (0.3–1.4) V potential range at 25 ± 2 °C. Charge/discharge tests were done using battery testing system CT-4008 (Neware Co., Shenzhen, China) in the (0.3–20) A g^−1^ current range, CV measurements were carried out on a BCS-805 potentiostat (Biologic Science Instruments, Seyssinet-Pariset, France) at a scan rate of (0.1–1) mV∙s^−1^.

Electrochemical impedance spectroscopy (EIS) measurements were conducted in 10 kHz–0.1 Hz frequency range at 1.4 V potential with 10 mV rms amplitude in a three-electrode electrochemical cell using a BCS-805 potentiostat (Biologic Science Instruments, Seyssinet-Pariset, France).

## 3. Results and Discussion

### 3.1. Physical Characterization

**Figure 1:**
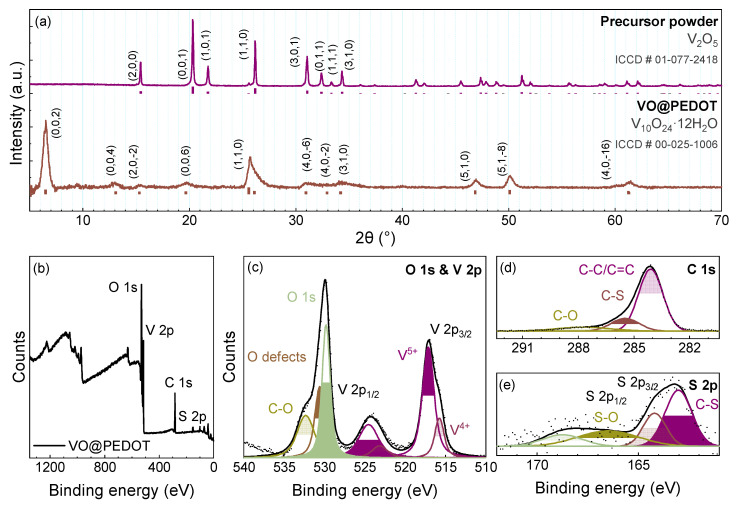
XRD patterns of the powders of the precursor V_2_O_5_ powder and VO@PEDOT composite (**a**); XPS survey spectrum of VO@PEDOT powder (**b**); XPS spectra of VO@PEDOT powder in O 1s and V 2p (**c**), C 1s (**d**), and S 2p regions (**e**).

The phase composition of the samples was determined by matching the XRD patterns (Figure 1a) with the ICCD data. The initial powder peaks are well-resolved and can be indexed to the orthorhombic V_2_O_5_ (ICCD #01-077-2418). The phase composition of the composite was determined by XRD analysis, which confirmed lamellar structure of vanadium oxide with monoclinic V_10_O_24_∙12H_2_O (ICDD #00-025-1006). The peaks are widened, indicating low crystallinity. The most intense peak of the (0,0,2) plane at 6.48° allows to estimate the interlayer distance in V_10_O_24_∙12H_2_O as 13.6 Å. This interlayer spacing is large enough for fast diffusion of intercalated zinc ions.

The XPS measurements were performed, and they showed the characteristic peaks of V, O, S, C elements in the survey spectrum (Figure 1b). The XPS spectra of V 2p (Figure 1c) show the changes occurring with the initial vanadium oxide powder upon the formation of the composite. For the initial V_2_O_5_ powder, the peaks at 524.4 eV and 526.0 eV for V 2p_3/2_ are related to V^5+^ and V^4+^, respectively, as per the available literature data [50]. The V 2p_3/2_–V 2p_1/2_ splitting value is 7.4 eV. The FWHM values for V 2p_3/2_ are ca. 1.4 eV, while V 2p_1/2_ peaks are broadened, as the FWHM values are 2.57 eV, which is a result of the Coster–Kronig effect [51]. The powder has a higher content of V^5+^, than of V^4+^, with the 5.9:1 ratio of the areas of the respective peaks. There are two O 1s peaks present: the one at 529.9 eV is typical [50] for oxygen in oxides and agrees well with the V 2p peaks. The second peak at a higher binding energy of 531.4 eV is related to the defective sites in the oxide crystal [52]. As the oxidative polymerization occurs when the composite forms upon the interaction of the initial V_2_O_5_ powder and EDOT monomer, the reduction of initial vanadium species is expected. XPS data confirms that assumption, as the ratio of V^5+^ to V^4+^ decreases for the composite. Further, the fitting of O 1s area for the composite sample reveals at least one additional peak at ~532.6 eV. This one is due to the aliphatic O-C-O bonds [53] in the PEDOT component of the composite. Additionally, this peak may relate to the oxygen within the structural water [54].

The XPS spectra of C 1s and S 2p (Figure 1d,e) additionally confirm the presence of PEDOT [53,55]. Specifically, the C 1s spectra contain three peaks: the most intense one at 284.3 eV marked as C-C/C=C corresponds either to single or double carbon–carbon bonds, i.e., either to the sp^3^ C-C bond and/or the sp^2^ C=C bond (since the corresponding binding energy difference is low, whereas the peak itself is broad, >1 eV FWHM) [56,57,58,59]. Even though there are cases where C-C/C=C peaks can be successfully distinguished [60], in our case the symmetricity was too high, as indicated by a tail mix of 99.9% for the fitted peak and is in line with the previously published research [61,62]. The peak at 285.6 eV is related to C-S bond, and the one at 287.7 eV is for C-O, which agrees with the O 1s spectra. The S 2p spectra contain the peaks typical for the presence of S-O (166.4 eV and 168.8 eV) and C-S bonds (163.1 eV and 164.4 eV) in the sample, which, along with the increase of signal-to-noise ratio as the temperature of the synthesis rises, further confirms the formation of the VO@PEDOT composite, yet the rise of the S-O peak also indicates partial PEDOT decomposition.

**Figure 2:**
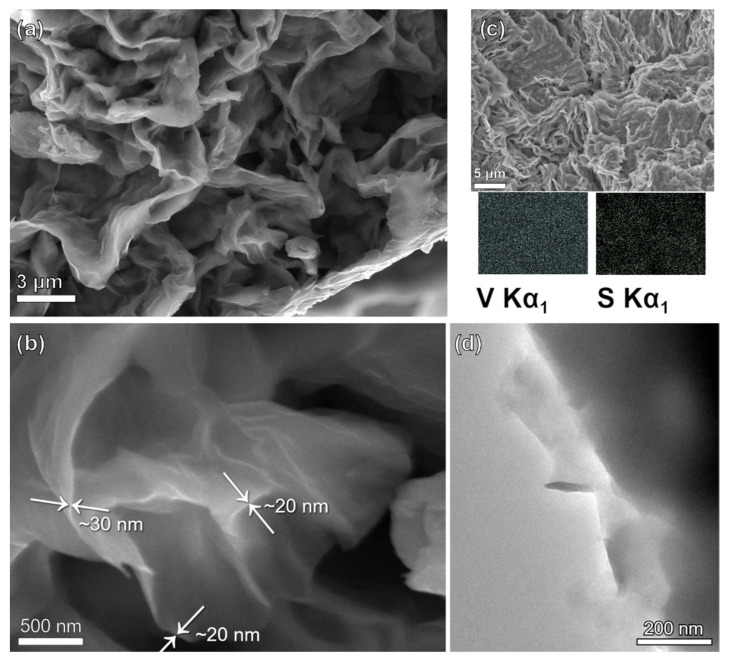
(**a**,**b**) SEM images of VO@PEDOT; (**c**) SEM image with the corresponding EDX mapping of V and S elements; (**d**) TEM image of VO@PEDOT.

The morphology of the obtained samples was studied by scanning electron microscopy (SEM). VO@PEDOT powder has layered nanosheets structure with thickness of about 30–80 nm, which are assembled into wavy agglomerated thicker layers up to 0.3–0.6 μm (Figure 2a,b). The EDX mapping (Figure 2c) for S and V elements confirms the uniformity of their distribution. In particular, sulfur distribution over surface of vanadium oxide grains supports the conclusion on the PEDOT shell. TEM image of as-prepared samples also exhibits thin-layer PEDOT coating of vanadium oxide nanostructure (Figure 2d).

**Figure 3:**
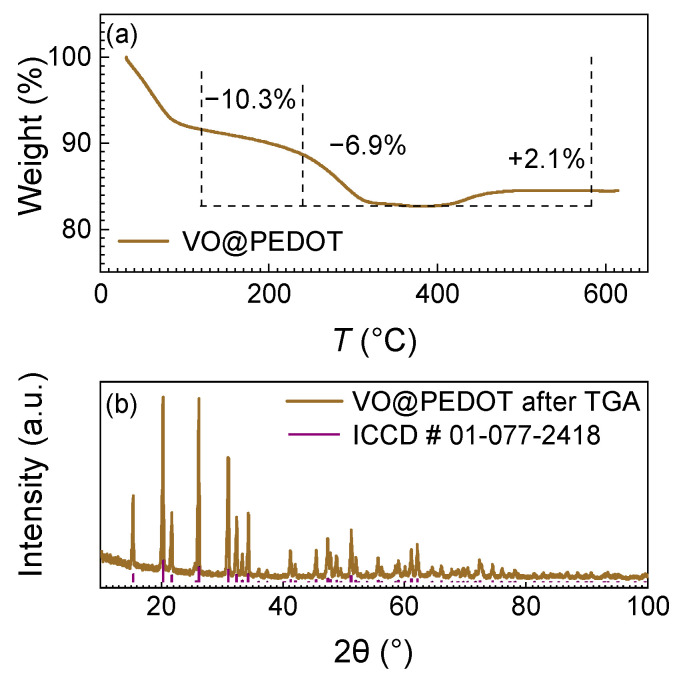
(**a**) TGA curve for VO@PEDOT and (**b**) its XRD pattern after TGA.

The thermogravimetric analysis in air (Figure 3a) allowed to determine PEDOT content in VO@PEDOT composite. The loss of water moisture causes the initial weight decrease, which goes through two stages: evaporation of free water, followed by the loss of interlayer water [54]. Then the decomposition of PEDOT occurs at ca. 120 °C, with its most intense evaporation starting at 240 °C. The decomposition completes at ~400 °C, and then the vanadium species in VO@PEDOT samples are oxidized to pristine orthorhombic V_2_O_5_, with an uptake of oxygen. This is confirmed by an XRD spectrum of the sample after the thermogravimetric analysis (Figure 3b). The content of PEDOT in VO@PEDOT sample is about 6.9%.

The increase of the weight also allows to estimate the ratios between V^4+^ and V^5+^ in the initial composite prior to full oxidation into V_2_O_5_. Considering the minimum weight as the sum of V_2_O_5_ and V_2_O_4_ weights, we may then estimate the V^5+^ to V^4+^ ratio as 3:1 for VO@PEDOT. This agrees well with the idea of partial reduction of initial V_2_O_5_ during the synthesis of nanocomposite.

For comparison the electrical conductivity of initial V_2_O_5_ and VO@PEDOT composite was determined using the impedance measurements with blocking electrodes. The conductivity of the VO@PEDOT sample (9.28⋅10^−4^ S cm^−1^) is four times as high as that of V_2_O_5_ (2.3⋅10^−4^ S cm^−1^). The earlier reported [49] conductivity value for an analogous material was lower by an order of magnitude: in our case the conductivity might be higher because of the slightly higher V^4+^ content in the sample.

### 3.2. Electrochemical Performance

Electrochemical performance of the electrode materials was evaluated by cyclic voltammetry (CV), galvanostatic charge–discharge (GCD), and electrochemical impedance spectroscopy (EIS) measurements.

**Figure 4:**
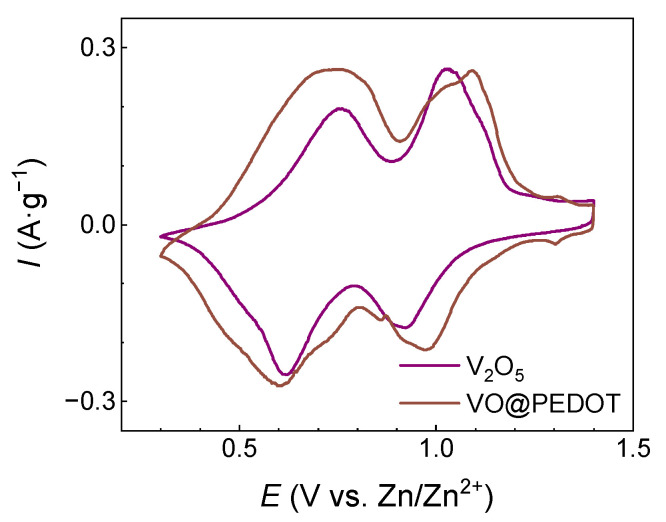
CV curves (10th cycle) of V_2_O_5_ and VO@PEDOT at a scan rate of 0.1 mV∙s^−1^.

Figure 4 shows the stable CV responses for coin cells with V_2_O_5_ and VO@PEDOT, that were obtained after the first ten cycles at a scan rate 0.1 mV∙s^−1^.

As seen from Figure 4, both CVs profiles demonstrate two pairs of peaks, located at potentials 0.76/0.61 V and 1.03/0.92 V for V_2_O_5_-based cathode and at potentials 0.75/0.60 V and 1.09/0.97 V for VO@PEDOT composite cathode. The area under peaks (normalized on the mass of active materials) for VO@PEDOT composite electrode was larger than for V_2_O_5_ electrode, which testifies the higher capacity value for composites.

**Figure 5:**
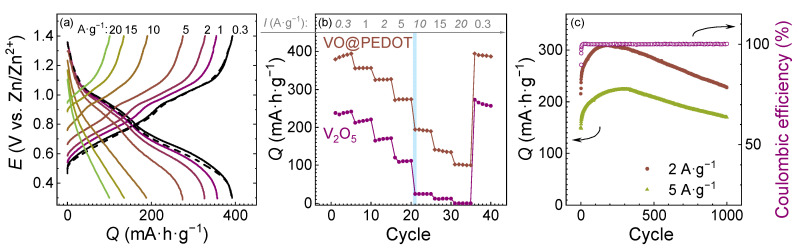
(**a**) GCD curves for VO@PEDOT at current densities from 0.3 A∙g^−1^ to 20 A∙g^−1^ (dashed line is at 0.3 A∙g^−1^ after all other currents); (**b**) C-rate performance of VO@PEDOT with comparison to initial V_2_O_5_ electrodes (after 10 cycles of activation); (**c**) GCD cycling performance of VO@PEDOT at 2 A∙g^−1^ and 5 A∙g^−1^.

Electrochemical performance of the electrode materials was investigated by galvanostatic charge/discharge (Figure 5). Charge/discharge profiles of VO@PEDOT demonstrated high initial capacity at low current densities (up to 390 mA∙h∙g^−1^ at 0.3 A∙g^−1^. The shape of charge and discharge curves is typical for vanadium-based cathodes with two skewed plateaus. It is clearly seen that polarization value is increased with the growth of current, so almost linear *E(Q)* dependence is observed at high current densities (5 A∙g^−1^ and more) indicating significant contribution of pseudocapacitive processes. C-rate capability of electrodes at different current densities is presented on Figure 5b. For VO@PEDOT composite material capacities of 357, 326, 274, 192, 137, and 101 mA∙h∙g^−1^ were obtained at current densities of 1, 2, 5, 10, 15, and 20 A∙g^−1^. In all cases these values are higher than for V_2_O_5_-based cathode which was previously activated at low current density. The capacities of V_2_O_5_ are 261, 217, 170, 110, 24, 11, and 0 mA∙h∙g^−1^ in the same current range. Nevertheless, for both electrode materials at 0.3 A∙g^−1^ after the cyclability tests the delivered capacities were the same as the initial ones, so the materials are not destructed during tests at high current densities. Cyclic stability of VO@PEDOT were recorded at 2 and 5 A∙g^−1^ over 1000 cycles (Figure 5c). At both current densities first a capacity increase and then a slight capacity drop were detected. At 2 A∙g^−1^, the maximum of capacity is achieved by the 175th cycle (310 mA∙h∙g^−1^). After that, the capacity decreases until the final value of 228 mA∙h∙g^−1^ is recorded. The capacity retention from the first cycle thus exceeds 100%, which means the material retains 73.5% of the maximum value.

At 5 A∙g^−1^, the specific capacity of VO@PEDOT electrode is 150 mA∙h∙g^−1^ initially, which rises to 225 mA∙h∙g^−1^ by the 370th cycle, and then decreases again to 170 mA∙h∙g^−1^ by the 1000th cycle. Again, the capacity retention exceeds 100% of the starting value, yet the final capacity is 75.5% of the maximum value. So, the VO@PEDOT electrode delivers the highest specific capacity values at every current density applied, likely due to facilitated kinetics of Zn^2+^ (de)intercalation.

**Figure 6:**
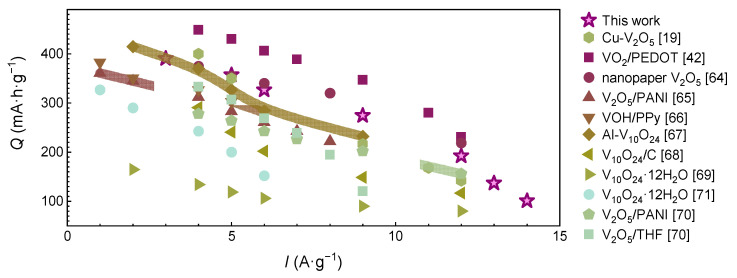
The comparison of the VO@PEDOT cathode with other V_2_O_5_-based cathodes for Zn-ion batteries [19,42,63,64,65,66,67,68,69,70,71].

These results are on par with the cyclic stability and rate capability of other state-of-the-art vanadium oxide-based composite materials [19,42,63,64,65,66,67,68,69,70], the functional properties of which are compared in Figure 6 and presented in the Table S1. As it can be seen, the performance of the materials in this work is on par with the state-of-the-art level for composite cathodes for AZIBs.

We should note that our synthesis procedure results in noticeable degree of reduction of V^5+^ into V^4+^, which means that structural transformations of the oxides occur, and PEDOT component is not the only beneficial contribution into the properties of the electrode materials. The oxide V_10_O_24_ is formed during the reductive synthesis may have a more advantageous structure for reversible and facile Zn^2+^ (de)insertion.

**Figure 7:**
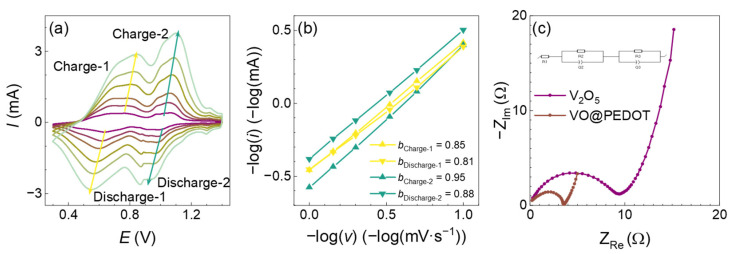
(**a**) CV curves for VO@PEDOT with different scan rates; (**b**) log *(i)* vs. log *(v)* curves of cathodic and anodic peaks of CV curves; (**c**) EIS spectra of the initial V_2_O_5_ and VO@PEDOT in 10 kHz to 100 mHz range at 1.4 V.

To estimate the contribution of diffusion-controlled and capacitive-controlled currents into the total measured currents, we performed CV measurements in the 0.3–1.4 V range at the scan rates from 0.1 mV∙s^−1^ to 1 mV∙s^−1^ (Figure 7a). The shape of the CVs at various scan rates is similar for VO@PEDOT electrodes with slight potential shifting. The dependence of the peak current (*i*) on the scan rate (*v*) can be represented via (1):

(1) $$i=a\cdot v^{b}$$

which can be represented in logarithmic form (2):

(2) $$\log\;\left(i\right)=b\cdot \log\;\left(v\right)+\;\log\;\left(a\right)$$

Here values *a* and *b* are constants. The constant *b* is usually in the 0.5 to 1.0 range, where the proximity to either 0.5 or 1.0 formally means that the current is controlled either by diffusion of the ions, or by pseudo-capacitive processes, respectively.

As seen from Figure 7b, for VO@PEDOT the *b* values indicate significant contribution of capacitive processes over all redox peaks, with 0.85/0.88 values for the leftmost pair of peaks and 0.95/0.81 for the rightmost one. The predominant pseudocapacitive control of current in the case of VO@PEDOT agrees well with the results reported for other V_2_O_5_-polymer composites [39,42,65,69,71].

The kinetics of the electrode processes was investigated by EIS measurements in three-electrode cell with 3 mol∙dm^−3^ ZnSO_4_ electrolyte solution. The impedance spectra (Figure 7c) were recorded in 10 kHz to 100 mHz frequencies range at 1.4 V after 10 GCD cycles at 0.1 A∙g^−1^. In the recorded spectra, the determined *R*_ct_ value was 9.2 Ω for V_2_O_5_, 2.2 Ω for VO@PEDOT. In agreement with reported enhanced capacity values, the VO@PEDOT electrode also has a lower *R*_ct_ value, which explains the facilitation of charge transport and hence can increase the capacity values of this material.

**Figure 8:**
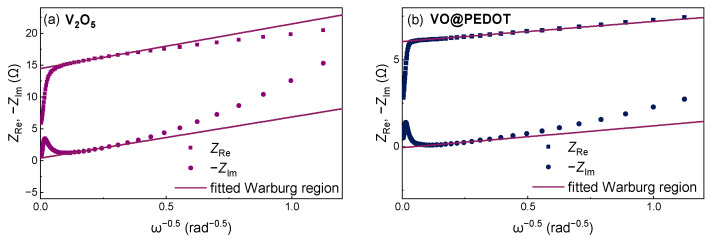
*Z*_Re_, −*Z*_Im_ vs. ω^−0.5^ plots obtained from the EIS spectra for V_2_O_5_ (**a**) and VO@PEDOT (**b**) electrodes. Linear fit was performed for the points in the Warburg region (i.e., at the straight line with a phase of 45° in Nyquist plot).

The appearance of Warburg type regions on EIS spectra with slope close to 45° (Figure 7c) indicates diffusion control of charge transport in the bulk of material. We calculated the Warburg constant values (σ_w_) for the studied systems via the slope of the *Z*_Re,_ −*Z*_Im_ vs. ω^−0.5^ linear dependencies (Figure 8). The value σ_w_ was 6.7 Ω·s^−0.5^ for the electrodes based on an unmodified V_2_O_5_, and lower value 1.2 Ω·s^−0.5^ for the VO@PEDOT electrodes. These values can further be used for the calculation of apparent diffusion coefficients via applicable formulae. For the standard inverse-square law between σ_w_ and ω^−0.5^, and considering the absence of other factors affecting the mass transport kinetics, the apparent diffusion coefficient for VO@PEDOT should be ca. 30 times as large as for V_2_O_5_. Thus, the obtained data confirm the faster ion intercalation process for VO@PEDOT electrode.

### 3.3. Mechanism Study

**Figure 9:**
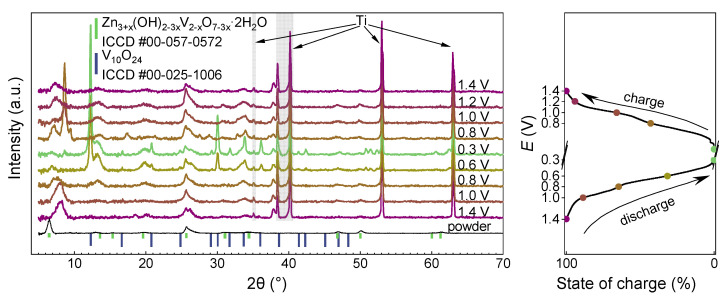
Ex situ XRD patterns of the VO@PEDOT electrode at various stages within the first discharge–charge cycle and the corresponding state-of-charge curve with the marked points of the potentials for XRD studies.

The ex situ XRD patterns (Figure 9) allow to elucidate the mechanism of Zn^2+^ intercalation into VO@PEDOT and the evolution of the composite structure. The spectra obtained at specific potentials during charge and discharge processes at 0.1 A∙g^−1^ allow to see the transformations in detail. All but the powder one spectra contain the peaks at 35.17°, 38.43°, 40.2°, 53.02°, and 62.96° of the titanium foil current collector (ICCD #00-044-1294). The peaks at 5–9° are related to the interlayer reflex (0,0,2) of V_10_O_24_∙12H_2_O (JCPDS #25-1006) [68]. Upon immersing the electrode into ZnSO_4_ at charging it to 1.4 V, the peak shifts to a more positive area. The shift of the reflection is due to water molecules and Zn^2+^ insertion into the interlayer space accompanied by electrostatic layers compression.

Then, upon the discharge to 0.6 V, well-resolved peaks emerge corresponding to the monoclinic zinc pyrovanadate Zn_3_(OH)_2_V_2_O_7_⋅2H_2_O (ICCD 00-057-0572) [31,72] while the V_10_O_24_∙12H_2_O-related peaks at 5–9° disappear. Further discharge to 0.3 V results in the increase of the peaks related to zinc pyrovanadate. As the pyrovanadate contains V^5+^ atoms in its structure, the discharge must include its reduction to V^4+^ or even (at least partially) to V^3+^, accompanied by Zn^2+^ or protons intercalation. This would make the exact formula differ from the matching XRD, e.g., Zn_3_V^4+^_2_O_7_ might describe the reduced state of the electrode material, with even further reduction forming a mixed valence compound Zn_3+*x*_V^4+^_2−2*x*_V^3+^_2+2*x*_O_7_, where 0 ≤ *x* ≤ 1. This condition is necessary to satisfy the conditions of charge accumulation and zinc storage, though it is worth noting that the XRD pattern still matches the pyrovanadate one in the fully reduced state, which might indicate the rigidity and stability of the obtained structure. Similar observations on reduction of electrode materials to pyrovanadate analogue structure have also been reported for V_2_O_5_ [73] and V_2_O_5_⋅nH_2_O/polypyrrole [65].

The reverse process of oxidation to 1.4 V results in gradual disappearance of pyrovanadate-related peaks. Narrow peaks and this high crystallinity of the sample at 0.3 V might indicate saturation of the structure by zinc ions until a stable well-ordered structure is obtained. The peaks related to the Zn_4_(OH)_6_SO_4_⋅nH_2_O (commonly referred to as zinc hydroxide sulfate (ZHS)) that have been observed in some other works [25,74,75], are absent in our case, which might indicate negligible content of ZHS in the prepared samples, as the existence of ZHS is heavily pH-dependent [76]. As noted in the work [77], when preparing ex situ electrodes, ZHS precipitate can be washed off and not be identified, therefore ex situ XRD data are not enough.

**Figure 10:**
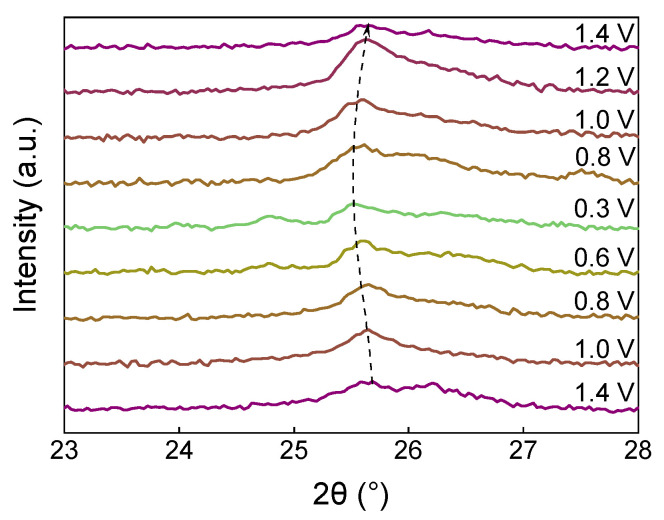
Ex situ XRD patterns of VO@PEDOT electrode upon the first discharge–charge cycle at 23–28°.

The reflection at 25.7° (Figure 10) related to the (111) plane of V_10_O_24_∙12H_2_O is preserved at each potential during charging and discharging. The transformation of the structure during the discharge shifts this peak to the lower angles, and then the peak returns to the initial position upon charging. Thus, the results of ex situ XRD show both the transformation of vanadium oxide structure within the VO@PEDOT composite and the reversibility of Zn^2+^ (de)intercalation. In addition to that, these results show that a novel phase emerges from V_10_O_24_∙12H_2_O upon recharging, while earlier data reported mainly on the intercalation-related peaks shifting [68,70,72]. This observation is likely caused by differences in morphology and presence of the conducting polymer within the cathode material.

**Figure 11:**
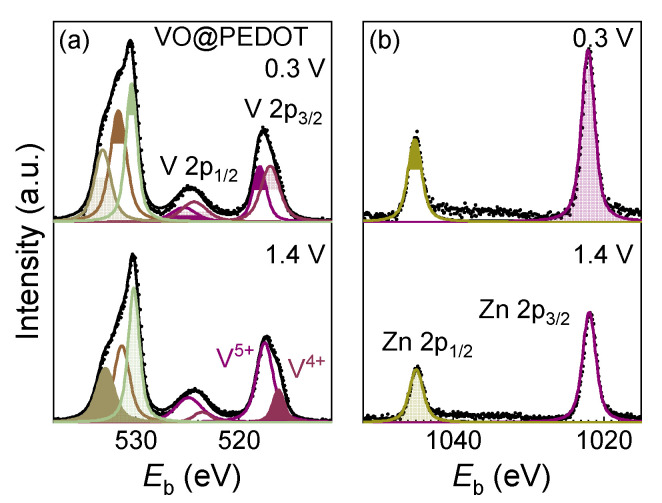
XPS spectra of V 2p (**a**) and Zn 2p (**b**) for VO@PEDOT electrode in the charged and discharged states.

The XPS spectra of O 1s and V 2p (Figure 11a), and Zn 2p (Figure 11b) obtained for VO@PEDOT electrodes in fully discharged (0.3 V) and fully charged (1.4 V) states after the first cycle show that the charged state resembles the initial VO@PEDOT powder (see also survey spectra in Figure S1). For the charged state, the V^5+^ peak prevails, and there is less V^4+^ (V^5+^:V^4+^ ratio is ca. 3.5:1). Yet the XPS of the sample in a discharged state shows that the amount of V^4+^ increases insignificantly. Additionally, the Zn 2p spectra of the identical electrodes in charged and discharged states show that the zinc content increases in the discharged state. Estimated ratios of Zn 2p_3/2_ to V 2p_3/2_ peaks in the charged and discharged states are 1.34 and 1.97, meaning that zinc content in the discharged state is 1.5 times higher than in the charged state, roughly in agreement with XRD data.

Moreover, EDX mapping of Zn and V elements for VO@PEDOT electrode in the charged and discharged states, as well as elemental composition analysis, showed that Zn to V atomic ratio is 1:1.27 in the discharged state and 1:2.47 in the charged state, which confirms the intercalation of Zn^2+^ into the material structure upon discharging. Ex situ SEM/EDX studies of the VO@PEDOT samples in the discharged state did not register any evidence of Zn_4_(OH)_6_SO_4_∙xH_2_O presence, which might indicate both its low generation and possibility of its dissolution upon samples preparation.

The dissolution of the electrodes containing orthorhombic V_2_O_5_ and VO@PEDOT composite in the 3 mol∙dm^−3^ ZnSO_4_ solution allowed to estimate the stability of the materials without any electrochemical processes running, which is essential for preserving the electrode materials without the cells for prolonged shelf and service life.

The results of AES-ICP analysis of vanadium content in 3 mol∙dm^−3^ ZnSO_4_ electrolyte (Table 1) show that VO@PEDOT composite dissolves at the lowest rate. After six months, the vanadium species content in VO@PEDOT solution is only 6.53 mg∙dm^−3^, whereas opposed to V_2_O_5_ solution with 57.8 mg∙dm^−3^ (one order higher) vanadium concentration.

Thus, the inclusion of PEDOT into the material structure further inhibits vanadium oxide dissolution in 3 M ZnSO_4_ at stationary conditions, which is also a key factor for improvement of electrochemical performance.

Such slow dissolution of VO@PEDOT composite is beneficial for its use in commercial devices, as it can preserve the material the longest in stationary conditions, which complements its splendid charge–discharge performance.

## 4. Conclusions

The vanadium oxide/poly(3,4-ethylenedioxythiophene) nanocomposite VO@PEDOT was successfully synthesized by a facile one-step microwave-assisted hydrothermal synthesis and tested as cathode in aqueous zinc-ion batteries. The initial composite obtained has layered nanosheets structure with thickness of about 30–80 nm, which are assembled into wavy agglomerated thicker layers up to 0.3–0.6 μm. The phase composition of vanadium oxide was determined by XRD analysis, which confirmed lamellar structure of V_10_O_24_∙12H_2_O with interlayer distance of about 13.6 Å.

The VO@PEDOT electrode shows an excellent electrochemical performance, reaching specific capacities of up to 390 mA∙h∙g^−1^ at 0.3 A∙g^−1^ and retains the specific capacity value of 100 mA∙h∙g^−1^ at a high current density of 20 A∙g^−1^.

In contrast with pristine V_2_O_5_ based cathode, an activation process usually observed for such materials was almost absent for VO@PEDOT. The AES analysis of vanadium content in 3 mol∙dm^−3^ ZnSO_4_ electrolyte showed the lowest rate of dissolution of VO@PEDOT. After six months, the vanadium species content in electrolyte solution is only 6.53 mg∙dm^−3^, as opposed to V_2_O_5_ solution with 57.8 mg∙dm^−3^ (one order higher) vanadium concentration.

The phase transformations during the cycling of VO@PEDOT electrodes at different degrees of charge/discharge were demonstrated by using ex situ XRD measurements. It allows us to conclude that the reversible zinc ion intercalation occurs in stable zinc pyrovanadate structures formed during discharge.

The outstanding electrochemical performance of VO@PEDOT composite cathodes confirm that pre-intercalation of conducting polymers into vanadium oxide structures is an advantageous way to improve functional characteristics of AZIB cathodes due to several factors: (i) Enhanced surface electronic conductivity and tight electrical contact over surface of active grains; (ii) increased diffusion of intercalated ions due to enlarged interlayer space; (iii) PEDOT essentially inhibits vanadium oxide dissolution in aqueous electrolytes.

## Figures and Tables

**Table 1:** Solubility results for V_2_O_5_ and VO@PEDOT in 3 mol∙dm^−3^ ZnSO_4_.

Cathode	Day 1, mg∙dm^−3^	Day 2, mg∙dm^−3^	Day 3, mg∙dm^−3^	Day 5, mg∙dm^−3^	Day 10, mg∙dm^−3^	Month 6, mg∙dm^−3^
V_2_O_5_	0.72	1.14	1.34	1.86	2.42	57.8
VO@PEDOT	0.60	0.70	0.99	1.56	1.87	6.53

## Data Availability

Data will be available upon request from the corresponding authors.

## Supplementary Materials

The following supporting information can be downloaded at: https://www.mdpi.com/article/10.3390/nano12213896/s1. Figure S1: Ex situ XPS survey spectra of VO@PEDOT electrode materials: (**a**) in the charged state; (**b**) in the discharged state. Table S1: The comparison of the VO@PEDOT cathode with other V_2_O_5_-based cathodes for Zn-ion batteries. The following references are also mentioned in Supplementary Materials: [37,39,41,42,63,64,65,66,67,68,69,70,71,78,79,80,81].
