# Supplementary material for: Vanadium Oxide-Poly(3,4-ethylenedioxythiophene) Nanocomposite as High-Performance Cathode for Aqueous Zn-Ion Batteries: The Structural and Electrochemical Characterization
Source: Nanomaterials (Basel). 2022 Nov 4;12(21):3896. doi: 10.3390/nano12213896 (PMC9654932; doi:10.3390/nano12213896)
Supplement: Supplementary file 1 [file nanomaterials-12-03896-s001.zip › nanomaterials-1966432-supplementary.pdf]

nanomaterials

Supplementary Materials

Vanadium Oxide-Poly(3,4-ethylenedioxythiophene) Nanocomposite as High-Performance Cathode for Aqueous Zn-Ion Batteries: The Structural and Electrochemical Characterization

Filipp S. Volkov [1], Svetlana N. Eliseeva [1], Mikhail A. Kamenskii [1], Alexey I. Volkov [1], Elena G. Tolstopjatova [1], Oleg V. Glumov [1], Lijun Fu [2] and Veniamin V. Kondratiev [1,*]

1 Institute of Chemistry, Saint Petersburg State University, 7/9 Universitetskaya Nab, 199034 Saint Petersburg, Russia
2 State Key Laboratory of Materials-Oriented Chemical Engineering, Nanjing Tech University, Nanjing 211816, China
* Correspondence: v.kondratev@spbu.ru

Citation: Volkov, F.S.; Eliseeva, S.N.; Kamenskii, M.A.; Volkov, A.I.; Tolstopjatova, E.G.; Glumov, O.V.; Fu, L.; Kondratiev, V.V. Vanadium Oxide-Poly(3,4-ethylenedioxythiophene) Nanocomposite as High-Performance Cathode for Aqueous Zn-Ion Batteries: The Structural and Electrochemical Characterization. *Nanomaterials* **2022**, *12*, 3896. https://doi.org/10.3390/nano12213896

Academic Editor: Wenbin Hu

Received: 27 September 2022
Accepted: 2 November 2022
Published: 4 November 2022

Publisher's Note: MDPI stays neutral with regard to jurisdictional claims in published maps and institutional affiliations.

Copyright: © 2022 by the authors. Licensee MDPI, Basel, Switzerland. This article is an open access article distributed under the terms and conditions of the Creative Commons Attribution (CC BY) license (https://creativecommons.org/licenses/by/4.0/).

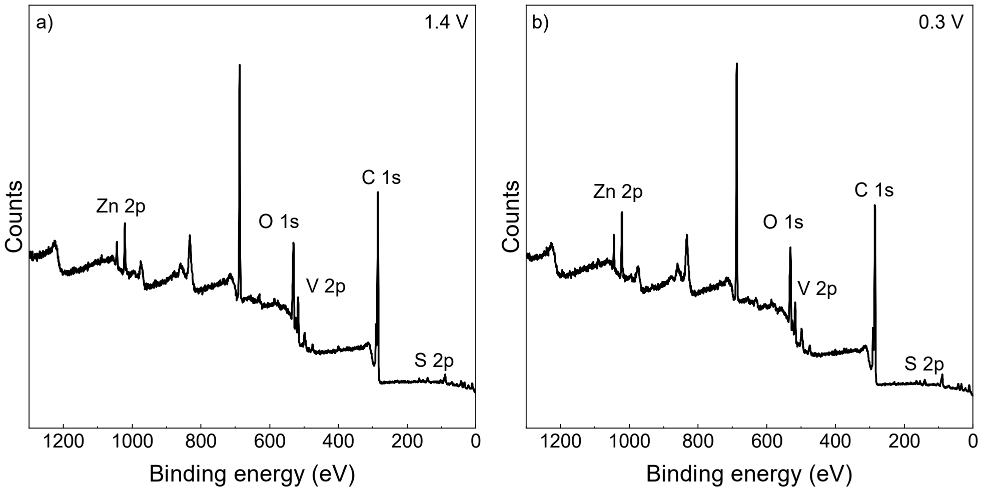

Figure S1. Ex situ XPS survey spectra of VO@PEDOT electrode materials: (**a**) in the charged state; (**b**) in the discharged state.

Table S1. The comparison of the VO@PEDOT cathode with other V_2O_5-based cathodes for Zn-ion batteries.

Material	Electrolyte	Specific capacity, mA·h·g^{-1} (current density)	Ref.
VO@PEDOT nanosheets	3 M $ZnSO_4$	390 (0.3 A·g^{-1})<br>357 (1 A·g^{-1})<br>274 (5 A·g^{-1})<br>192 (10 A·g^{-1})	This work
Al-$V_{10}O_{24}$ nanobelts	3 M $Zn(CF_3SO_3)_2$	370 (0.5 A·g^{-1})<br>327 (1 A·g^{-1})<br>232 (5 A·g^{-1})	[1]
$V_{10}O_{24}$/C nanosheets	3 M $Zn(CF_3SO_3)_2$	290.5 (0.5 A·g^{-1})<br>241 (1 A·g^{-1})<br>148.6 (5 A·g^{-1})<br>116.7 (10 A·g^{-1})	[2]
$V_{10}O_{24}·12H_2O$ nanobelts	3 M $Zn(CF_3SO_3)_2$	134 (0.5 A·g^{-1})<br>119 (1 A·g^{-1})<br>90 (5 A·g^{-1})<br>80 (10 A·g^{-1})	[3]
$V_{10}O_{24}·12H_2O$ layered	2 M $ZnSO_4$	243 (0.5 A·g^{-1})<br>200 (1 A·g^{-1})<br>152 (2 A·g^{-1})	[4]
V_2O_5/PANI nanocomposite	3 M $Zn(CF_3SO_3)_2$	312 (0.5 A·g^{-1})<br>283 (1 A·g^{-1})<br>201 (6 A·g^{-1})	[5]
V_2O_5/PANI crisscrossed leaf-like	3 M $Zn(CF_3SO_3)_2$	278 (0.5 A·g^{-1})<br>264 (1 A·g^{-1})<br>202 (5 A·g^{-1})<br>156 (10 A·g^{-1})	[6]
V_2O_5/THF nanobelts	3 M $Zn(CF_3SO_3)_2$	333 (0.5 A·g^{-1})<br>307 (1 A·g^{-1})<br>121 (5 A·g^{-1})	[6]
PANI-V_2O_5 nanosheets (100 °C)	3 M $Zn(CF_3SO_3)_2$	360 (0.5 A·g^{-1})<br>342 (1 A·g^{-1})<br>272 (5 A·g^{-1})<br>216 (10 A·g^{-1})	[7]
PANI-V_2O_5 nanosheets (120 °C)	3 M $Zn(CF_3SO_3)_2$	297 (0.5 A·g^{-1})<br>276 (1 A·g^{-1})<br>199 (5 A·g^{-1})<br>133 (10 A·g^{-1})	[7]
$(PANI)_x$ V_2O_5 nanosheets	3 M $Zn(CF_3SO_3)_2$	295 (0.5 A·g^{-1})<br>250(1 A·g^{-1})<br>190 (5 A·g^{-1})	[8]
PANI/V_2O_5	2 M $Zn(CF_3SO_3)_2$	~340 (0.5 A·g^{-1})	[9]

aggregated flakes		~330 (1 $A\cdot g^{-1}$)<br>~290 (5 $A\cdot g^{-1}$)<br>235 (20 $A\cdot g^{-1}$)	
PANI/V_2O_5 sponge-like particles	2 M $Zn(CF_3SO_3)_2$	380 (0.5 $A\cdot g^{-1}$)<br>340 (1 $A\cdot g^{-1}$)<br>247 (2 $A\cdot g^{-1}$)	[10]
PPy/VOH nanosheets	3 M $Zn(CF_3SO_3)_2$	327 (0.5 $A\cdot g^{-1}$)<br>303 (1 $A\cdot g^{-1}$)<br>281 (2 $A\cdot g^{-1}$)	[11]
V_2O_5@PPy	2 M $ZnSO_4$	186.4 (0.5 $A\cdot g^{-1}$)<br>101.8 (1 $A\cdot g^{-1}$)<br>65.3 (5 $A\cdot g^{-1}$)	[12]
V_2O_5@PEDOT monolithic grains	3 M $Zn(CF_3SO_3)_2$	360 (1 $A\cdot g^{-1}$)<br>280 (5 $A\cdot g^{-1}$)<br>197 (10 $A\cdot g^{-1}$)	[13]
PEDOT-VO 3D flowers	3 M $Zn(CF_3SO_3)_2$	~325 (0.5 $A\cdot g^{-1}$)<br>~290 (5 $A\cdot g^{-1}$)<br>~275 (10 $A\cdot g^{-1}$)	[14]
V_2O_5@PEDOT/CC core/shell nanosheet arrays	2.5 M $Zn(CF_3SO_3)_2$	282.2 (0.5 $A\cdot g^{-1}$)<br>274.5 (1 $A\cdot g^{-1}$)<br>254.1 (5 $A\cdot g^{-1}$)<br>240.2 (10 $A\cdot g^{-1}$)	[15]
VO_2/PEDOT nanobelts	3 M $Zn(CF_3SO_3)_2$	448.5 (0.5 $A\cdot g^{-1}$)<br>430.4 (1 $A\cdot g^{-1}$)<br>347.2 (5 $A\cdot g^{-1}$)<br>231.2 (10 $A\cdot g^{-1}$)	[16]
V_2O_5 nanopaper	2 M $ZnSO_4$	375 (0.5 $A\cdot g^{-1}$)<br>355 (1 $A\cdot g^{-1}$)<br>290 (6 $A\cdot g^{-1}$)<br>219 (10 $A\cdot g^{-1}$)	[17]

References

1. Li, Q.; Wei, T.; Ma, K.; Yang, G.; Wang, C. Boosting the Cyclic Stability of Aqueous Zinc-Ion Battery Based on Al-Doped V10O24·12H2O Cathode Materials. *ACS Appl. Mater. Interfaces* **2019**, *11*, 20888–20894, doi:10.1021/acsami.9b05362.
2. Wu, W.; Wang, S.; Zhang, C.; Hou, S.; Zhang, L. Facile and Scalable Synthesis of 3D Structures of V10O24·12H2O Nanosheets Coated with Carbon toward Ultrafast and Ultrastable Zinc Storage. *ACS Appl. Mater. Interfaces* **2021**, *13*, 18704–18712, doi:10.1021/acsami.1c00749.
3. Wei, T.; Li, Q.; Yang, G.; Wang, C. High-rate and durable aqueous zinc ion battery using dendritic V10O24·12H2O cathode material with large interlamellar spacing. *Electrochim. Acta* **2018**, *287*, 60–67, doi:10.1016/j.electacta.2018.08.040.
4. Liu, W.; Dong, L.; Jiang, B.; Huang, Y.; Wang, X.; Xu, C.; Kang, Z.; Mou, J.; Kang, F. Layered vanadium oxides with proton and zinc ion insertion for zinc ion batteries. *Electrochim. Acta* **2019**, *320*, doi:10.1016/j.electacta.2019.134565.
5. Du, Y.; Wang, X.; Man, J.; Sun, J. A novel organic-inorganic hybrid V2O5@polyaniline as high-performance cathode for aqueous zinc-ion batteries. *Mater. Lett.* **2020**, *272*, 127813, doi:10.1016/j.matlet.2020.127813.
6. Yan, H.; Ru, Q.; Gao, P.; Shi, Z.; Gao, Y.; Chen, F.; Chi-Chun Ling, F.; Wei, L. Organic pillars pre-intercalated V4+-V2O5·3H2O

nanocomposites with enlarged interlayer and mixed valence for aqueous Zn-ion storage. *Appl. Surf. Sci.* **2020**, *534*, 147608, doi:10.1016/j.apsusc.2020.147608.

7. Chen, S.; Li, K.; Hui, K.S.; Zhang, J. Regulation of Lamellar Structure of Vanadium Oxide via Polyaniline Intercalation for High-Performance Aqueous Zinc-Ion Battery. *Adv. Funct. Mater.* **2020**, *30*, 1–11, doi:10.1002/adfm.202003890.
8. Li, R.; Xing, F.; Li, T.; Zhang, H.; Yan, J.; Zheng, Q.; Li, X. Intercalated polyaniline in V2O5 as a unique vanadium oxide bronze cathode for highly stable aqueous zinc ion battery. *Energy Storage Mater.* **2021**, *38*, 590–598, doi:10.1016/j.ensm.2021.04.004.
9. Yin, C.; Pan, C.; Liao, X.; Pan, Y.; Yuan, L. Regulating the Interlayer Spacing of Vanadium Oxide by In Situ Polyaniline Intercalation Enables an Improved Aqueous Zinc-Ion Storage Performance. *ACS Appl. Mater. Interfaces* **2021**, *13*, 39347–39354, doi:10.1021/acsami.1c09722.
10. Wang, Z.; Tang, X.; Yuan, S.; Bai, M.; Wang, H.; Liu, S.; Zhang, M.; Ma, Y. Engineering Vanadium Pentoxide Cathode for the Zero-Strain Cation Storage via a Scalable Intercalation-Polymerization Approach. *Adv. Funct. Mater.* **2021**, *31*, 2100164, doi:10.1002/adfm.202100164.
11. Feng, Z.; Sun, J.; Liu, Y.; Jiang, H.; Hu, T.; Cui, M.; Tian, F.; Meng, C.; Zhang, Y. Polypyrrole-intercalation tuning lamellar structure of V2O5·nH2O boosts fast zinc-ion kinetics for aqueous zinc-ion battery. *J. Power Sources* **2022**, *536*, 231489, doi:10.1016/j.jpowsour.2022.231489.
12. Dong, R.; Zhang, T.; Liu, J.; Li, H.; Hu, D.; Liu, X.; Xu, Q. Mechanistic Insight into Polypyrrole Coating on V2O5 Cathode for Aqueous Zinc-Ion Battery. *ChemElectroChem* **2022**, *9*, doi:10.1002/celc.202101441.
13. Yao, Z.; Wu, Q.; Chen, K.; Liu, J.; Li, C. Shallow-layer pillaring of a conductive polymer in monolithic grains to drive superior zinc storage: Via a cascading effect. *Energy Environ. Sci.* **2020**, *13*, 3149–3163, doi:10.1039/d0ee01531h.
14. Li, S.; Wei, X.; Wu, C.; Zhang, B.; Wu, S.; Lin, Z. Constructing Three-Dimensional Structured V2O5/Conductive Polymer Composite with Fast Ion/Electron Transfer Kinetics for Aqueous Zinc-Ion Battery. *ACS Appl. Energy Mater.* **2021**, *4*, 4208–4216, doi:10.1021/acsaem.1c00573.
15. Xu, D.; Wang, H.; Li, F.; Guan, Z.; Wang, R.; He, B.; Gong, Y.; Hu, X. Conformal Conducting Polymer Shells on V2O5 Nanosheet Arrays as a High-Rate and Stable Zinc-Ion Battery Cathode. *Adv. Mater. Interfaces* **2019**, *6*, 1–8, doi:10.1002/admi.201801506.
16. Liu, X.; Xu, G.; Zhang, Q.; Huang, S.; Li, L.; Wei, X.; Cao, J.; Yang, L.; Chu, P.K. Ultrathin hybrid nanobelts of single-crystalline VO2 and Poly(3,4-ethylenedioxythiophene) as cathode materials for aqueous zinc ion batteries with large capacity and high-rate capability. *J. Power Sources* **2020**, *463*, doi:10.1016/j.jpowsour.2020.228223.
17. Li, Y.; Huang, Z.; Kalambate, P.K.; Zhong, Y.; Huang, Z.; Xie, M.; Shen, Y.; Huang, Y. V2O5 nanopaper as a cathode material with high capacity and long cycle life for rechargeable aqueous zinc-ion battery. *Nano Energy* **2019**, *60*, 752–759, doi:10.1016/j.nanoen.2019.04.009.
